# Cancer-associated fibroblast heterogeneity and its role in reshaping immunotherapy in solid cancers: potential strategies and clinical promise

**DOI:** 10.3389/fcell.2026.1761070

**Published:** 2026-03-10

**Authors:** Peng Zu, Zhanwu Yu

**Affiliations:** Department of Thoracic Surgery, Cancer Hospital of China Medical University, Liaoning Cancer Hospital & Institute, Cancer Hospital of Dalian University of Technology, Shenyang, China

**Keywords:** cancer-associated fibroblast, heterogeneity, immunotherapy, solid cancers, TME

## Abstract

Cancer-associated fibroblasts (CAFs) are important components of the solid tumour microenvironment (TME). CAFs have long been regarded as major promoters of the malignant progression of tumours and are widely recognized for their strong secretory activity and direct effect on the malignant ability of tumour cells. Recently, studies have found extensive crosstalk between CAFs and tumour immunity. CAFs constitute a highly heterogeneous group, and continuous studies have shown that their subpopulations have unique functions and can be widely involved in tumour immune regulation and immunotherapy. However, the specific mechanisms still need to be further revealed. In this paper, we focused on the interactions between CAFs subpopulations and immune cells in the TME and summarized the interactions between CAFs and immune cells from multiple perspectives to provide new insights for antitumour immunotherapy in solid tumours.

## 1 Introduction

Cancer is currently the leading cause of disease-related death. Despite the development of various strategies and drugs to prevent tumour progression, patient mortality remains high. At present, improving cancer treatment and proposing new targets are crucial for future clinical treatment (Maia et al., 2023; Siegel et al., 2022). The importance of the immune system during the development of cancers is widely recognized, and targeting immune system has triggered a new revolution in antitumour immunotherapy. Among the different immunotherapy strategies, the most intensively studied are immune checkpoint inhibitors (ICIs) targeting CTLA-4 and PD-1 receptors, and ICIs greatly improve antitumour immunotherapy efficiency especially in several solid tumours, such as lung cancer (Donini et al., 2023; Okuyama et al., 2023; Qin et al., 2019; Zhang et al., 2023a). Although this strategy significantly improved patient outcomes, many shortcomings still exist. For example, its effectiveness remains low and has been validated only in a subset of tumours. Notably, the current theory suggests that immune efficacy may be modulated by the tumour microenvironment (TME). CAFs are among the most important components of the TME and are closely related to tumour therapy and other aspects, including treatment response, and their importance in modulating antitumour immunity has been widely demonstrated (Zhang et al., 2023a; Brichkina et al., 2023; Papait et al., 2022) (Table 1). Therefore, understanding the interaction between CAFs and immune cells is necessary to further improve the effectiveness of immunotherapy.

**TABLE 1:** Characteristics and functions of heterogeneous populations of CAFs.

Cancers	Subtypes	Characteristics	Functions	References
PDAC	myCAFs	α-SMA, TAGLN, MYL9, TPM1/2, HOPX, POSTN, MMP11, AGTR1, AT1	ECM remodelling	Elyada et al. (2019)
	iCAFs	PDGFRα, CFD, LMNA, DPT, CXCL1, CXCL2, CCL2	Immune suppression and Chemoresistance	Elyada et al. (2019)
	apCAFs	MHC class II, H2-Aa, H2-Ab1, CD74, Col1a1, Col1a2, DCN, PDPN, CD239, CD321	Antigen-present and Immune modulation	Elyada et al. (2019)
	POSTN+ CAF	POSTN	Mainly promoting invasion, reduced tumour-promoting activity, good/intermediate prognosis	Neuzillet et al. (2019)
	—	MYH11, α-SMA, Vimentin	Lymph node metastasis, intermediate prognosis	Neuzillet et al. (2019)
	—	PDPN	Immune promotion, good prognosis	Neuzillet et al. (2019)
Breast cancer	myCAFs	Dcn, Lum, Vcan, Col14a1, Fbln1, Fbln2, Smoc, Lox, Loxl1	Angiogenesis, EMT, immune response	Bartoschek et al. (2018)
	iCAFs	Ly6c1, CLEC3B, HAS1, DPT, COL14A1	Angiogenesis, immune evasion, chemoresistance	Bartoschek et al. (2018)
	apCAFs	CD74, H2-Aa, H2-Ab1, H2-Eb1, KRT18, FSP1	Antigen presentation, immune modulation	Bartoschek et al. (2018)
	vCAFs	Notch3, Epas1, Col18a1, Nr2f2, Nidogen-2	Angiogenesis	Bartoschek et al. (2018)
	mCAFs	Fibulin-1, PDGFRα, CXCL14	Immune regulation	Bartoschek et al. (2018)
	dCAFs	Scrg1, Sox9, Sox10	—	Bartoschek et al. (2018)
	CD10 + GPR77+	CD10, GPR77	Chemoresistance	Su et al. (2018)
OSCC	—	HA, MMPs	Tumour invasion, immunosuppression	Costea et al. (2013)
	—	TGF-β	Tumour migration	Costea et al. (2013)
Colorectal cancer	—	MMP2, DCN, αFAP, COL1A2	ECM remodelling	Li et al. (2017)
	—	α-SMA, ACTA2, TAGLN, PDGFA	Not reported	Li et al. (2017)
HGSOC	—	CD29, FAP, αSMA, FSP1, PDGFRβ, CXCL12β	Tumour proliferation, immune suppression	Givel et al. (2018)
	—	Not reported	—	Givel et al. (2018)
	—	CD29, FSP1, PDGFRβ	—	Givel et al. (2018)
	—	CD29, αSMA, FSP1, PDGFRβ	Tumour proliferation	Givel et al. (2018)
ICC	iCAFs	Lrat, Reln, Rgs5	Tumour proliferation, immune suppression	Affo et al. (2021)
	myCAFs	Col1a1, Acta2, Col8a1, Col15a1, Crlf1, Fbn2	Tumour invasion, proliferation	Affo et al. (2021)
	mesCAFs	Msln, Upk1b, Upk3b, Gpm6a	—	Affo et al. (2021)

## 2 Regulatory networks of CAF activation

### 2.1 Epigenetic modifications

The difference between NFs and CAFs is considered to be key for improving tumour treatment strategies. It is generally believed that the CAF genome is relatively stable and that transcriptional regulation plays an important role in reprogramming. Moreover, transcription factors PRRX1 and SOX2 are closely related to the acquisition of CAF phenotype in fibroblasts (Halperin et al., 2022; Kasashima et al., 2021; Lee et al., 2022). DNA methylation and histone methylation/acetylation are important processes in the transcriptional modification of transcription factors. Many studies have found that CAFs are similar to tumour cells, and both of them have abnormal DNA methylation patterns, which also indicates that abnormal transcriptional modification is the key modification link between NFs and CAFs (Zhang et al., 2023b). Although DNA methylation levels in CAFs vary across different tumour types, the overall DNA methylation level of CAFs is low in most malignant tumours, and this is accompanied by DNA hypermethylation in some CAFs (Fiori et al., 2019; Mao et al., 2021). Enhancers, as members of cis-regulatory elements, play key roles in regulating transcription factor function at a distance and in specific gene expression programs that determine cell type or cell state. During CAF activation, histone H3 lysine 27 acetylation (H3K27ac) and histone H3 lysine 4 mono-methylation (H3K4me1) were detected in CAFs.

### 2.2 Transcription-related regulation

#### 2.2.1 HIF

**FIGURE 1:**
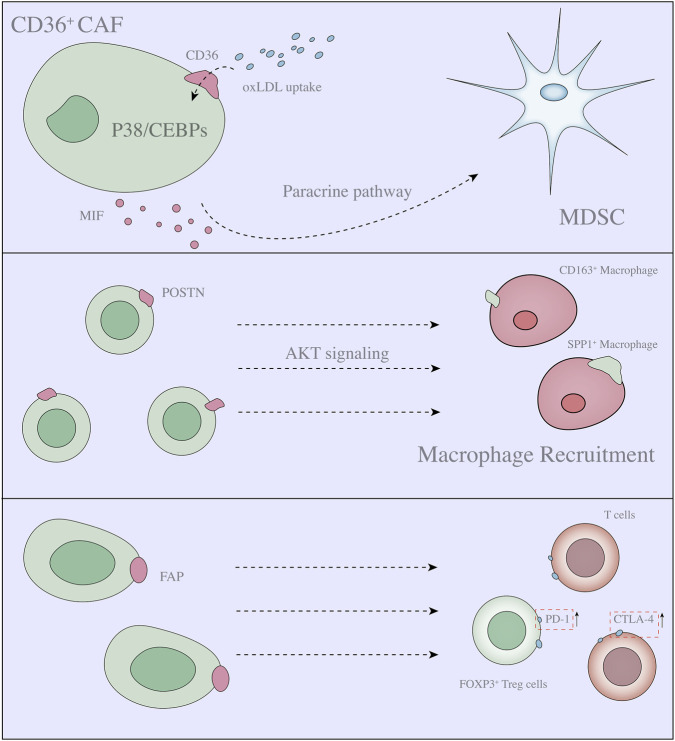
Mechanisms of CAF activation at the transcriptional level.

HIF is the main molecule of hypoxia-related transcription factors, which can reduce the oxygen consumption of cells by regulating glycolysis and mitochondrial function, allowing cells to adapt to the hypoxic microenvironment (Figure 1). Many studies have shown that HIF plays an important role in CAF activation (Denko, 2008; Ratcliffe, 2013). Compared with that in normal fibroblasts (NFs), HIF-1α expression is significantly increased in lung cancer CAFs. When HIF-1α is overexpressed in NFs, NFs rapidly transform into CAFs, and activated CAFs promote lung cancer progression through CCL-5 signalling (Zhang et al., 2021). Mechanistically, CAFs have higher glycolysis levels. HIF-1α-mediated activation of CAFs induces metabolic reprogramming in breast cancer by increasing glucose uptake, increasing the protein expression of glycolytic enzymes, and inhibiting oxidative phosphorylation through NDUFA4 to promote glycolysis (Becker et al., 2020; Zhang et al., 2015). The shift in metabolism may be critical in forcing NFs to transition into CAFs. Interestingly, the effect of HIF-1α on glycolysis is not limited to CAFs. CAF-derived exosomes can participate in glycolysis by regulating HIF-1α expression in tumour cells (Liu et al., 2021). This implies that the role of HIF-1α may be conserved; in addition to its involvement in CAF activation, HIF-1α can also induce HIF-1α expression in target cells, thereby promoting tumour progression. Therefore, targeted therapy against HIF-1α has become a promising treatment strategy. Metformin, a clinically used drug, prevents crosstalk between CAF and tumours by inhibiting HIF-1α, ultimately reducing tumour malignancy (Shao et al., 2020). However, it should be noted that CAF activation could be inhibited under chronic hypoxia (Madsen et al., 2015). Therefore, the degree and duration of hypoxia need to be considered in the context of targeted therapy.

#### 2.2.2 p53

As a tumour suppressor gene, p53 has been shown to play an important regulatory role in CAFs. Studies have shown that the abnormal function of p53 differs between CAFs and NFs and that p53 mutations play a key role in CAF activation, influencing their secretome (Arandkar et al., 2018). In addition to altering p53 function to affect CAF activation, p53 knockout also affects CAF activation and proliferation. p53 knockout in the stroma strongly promotes CAF proliferation, whereas normal epithelial cells are not affected. In addition, p53-null CAFs effectively block the immune response (Wu et al., 2022). Interestingly, analysis of 32 lung cancer tissues, including sectioning and staining, revealed that p53 expression was highly consistent with that in tumour cells, even after cisplatin treatment. The reason for this phenomenon is not clear. However, CAFs have strong secretory properties, and crosstalk between the stoma and tumour needs to be considered. This communication may also exist between CAFs and immune cells.

#### 2.2.3 TWIST1

TWIST1 can activate CAFs and remodel the ECM. Normal fibroblasts transfected with TWIST1 exhibit characteristics of CAFs and increase the stiffness of the ECM. Another study reported that IL-6 induced TWIST1 expression in NFs and promoted their transdifferentiation into CAFs by activating STAT3 and that CXCL12 is a downstream target of TWIST1 (Garcia-Palmero et al., 2016). In contrast, CXCL12/C-X-C chemokine receptor (CXCR) 4 signalling promotes EMT through the AKT/TWIST1/MMP1/E-cadherin axis. These findings suggest the importance of TWIST for CAF activation (Zhu et al., 2019).

#### 2.2.4 ZEB1

ZEB1 has long been regarded as a key factor in epithelial‒mesenchymal transition (EMT) (Liu et al., 2008). Increased ZEB1 expression promotes CAF-mediated invasive structure formation and endows CAFs with potent secretory properties (Bota-Rabassedas et al., 2021). This is the key feature of CAF activation (Fang et al., 2023). Similarly, ZEB1 plays an important role in EMT in cholangiocarcinoma, where ZEB1 activates cell dedifferentiation characteristics and promotes paracrine signalling in mesenchymal cells (Lobe et al., 2021). These results initially define the key role of ZEB in CAF activation in tumours.

## 3 CAF heterogeneity and immune regulation

Differences in CAF function, origin, and molecular marker diversity have led to the artificial classification of CAFs into several subtypes, which only partially overlap. The heterogeneity of CAFs has been known for many years through flow cytometry analysis (Ikenaga et al., 2010). With the development of single-cell technology, several heterogeneous groups of tumours have been identified (Bachem et al., 2005; Ohlund et al., 2017). Myofibroblastic CAFs, antigen-presenting CAFs (apCAFs), and inflammatory CAFs (iCAFs) are well-known subgroups with distinct functions (Huang et al., 2022; Ma et al., 2023; Zhan et al., 2025). For example, iCAFs play important roles in inflammatory responses, immunosuppression, and angiogenesis, whereas myCAFs mainly perform the function of matrix remodelling and can also affect tumour angiogenesis (Ma et al., 2023; Zhan et al., 2025). However, the function of apCAFs mainly involves the regulation of antigen presentation and T-cell activation (Huang et al., 2022; Song et al., 2025). In subsequent studies, as technology advanced, especially in patients with immunotherapy resistance, drug resistance and metastasis, several unique CAF subgroups were identified that possess unique capabilities during tumour progression (Elyada et al., 2019; Dominguez et al., 2020; Zhou et al., 2024). THBS2+ CAFs induce COL8A1-mediated AKT signalling activation to promote EMT, leading to oxaliplatin resistance in colon cancer cells *in vitro* (Zhou et al., 2024). Interestingly, these subtypes have various unknown specific functions. However, little is known about the function of CAF subsets (Lavie et al., 2022; Sugimoto et al., 2006). Owing to the differences in experimental techniques and methods in different studies, it is difficult to define CAFs using a single or a few marker molecules. In this review, we analysed mainly the potential therapeutic strategies involving CAF subsets by summarizing the mechanisms of different studies on the regulation of immune cell function. CAFs can directly modulate immune cell function in TME, thereby promoting tumour growth, immune escape and metastasis. This activity is largely dependent on the accumulation of inhibitory myeloid T cells and regulatory T cells (Tregs) by cytokines and chemokines, the inhibition of antigen presentation by dendritic cells, and the promotion of macrophage and T-cell polarization (Monteran and Erez, 2019). However, the regulatory effects of heterogeneous CAF populations on immune cells have not been fully elucidated. Here, we summarize the current research to provide new insights for subsequent treatment (Table 2: Markers of CAFs in different tumours (Elyada et al., 2019; Neuzillet et al., 2019; Bartoschek et al., 2018; Su et al., 2018; Costea et al., 2013; Li et al., 2017; Givel et al., 2018; Affo et al., 2021)). Multiple subpopulations of CAFs constitute a complex survival niche in TME, and CAFs communicate with immune cells to generate various cell types.

**TABLE 2:** Markers of CAFs in different tumours.

Subsets	Markers	Cancer type	References
iCAFs	PDPN, lL-6, α-SMA (low), LIF	Pancreatic cancer	Ohlund et al. (2017)
FAP+ CAFs (PanIN region)	FAP+, αSMA (low), CD34^+^, PDGFRα+, CD45−	Pancreatic cancer	Feig et al. (2013)
FAP+ CAFs (PDA region)	FAP+, αSMA+, CD34−, PDGFRα+, CD45−	Pancreatic cancer	Feig et al. (2013)
ZIP1+ CAFs	ZIP1, NOTCH2	Lung cancer	Ni et al. (2022)
HMGB2+ CAFs	HMGB2, CDK1, CCNA2, CCNB1, CCNB2	Lung cancer	Ni et al. (2022)
metCAFs	COL3A1+, NNMT+	Lung cancer	Ni et al. (2022)
myCAFs	α-SMA (high), FAP+, CTGF+, TNC+, TAGLN+	Pancreatic ductal adenocarcinoma	Biffi et al. (2019)
apCAFs	PDPN, SAA3, MHC-II, CA74	Pancreatic ductal adenocarcinoma	Elyada et al. (2019)
Secretory CAFs	FAP, α-SAM, CD10	Pancreatic ductal adenocarcinoma	Awaji et al. (2020)
CAF-S1	FAP (high), α-SMA (high), PDPN(high), PDGFRβ(high)	Breast cancer	Pelon et al. (2020)
CAF-S2	FAP-, CD29−, α-SMA−, PDPN(low), PDGFRβ(low)	Breast cancer	Pelon et al. (2020)
CAP-S3	FAP (low), α-SMA (low), PDPN(low), PDGFRβ(low)	Breast cancer	Pelon et al. (2020)
CAF-S4	FAP (low), CD29 (high), α-SMA (high), PDPN(low)	Breast cancer	Pelon et al. (2020)
vCAFs	Nidogen-2, CD31, NOTCH3, EPAS1, COL18A1, NR2F2	Breast cancer	Bartoschek et al. (2018)
mCAFs	PDGFRα, Fibulin-l	Breast cancer	Bartoschek et al. (2018)
cCAFs	Not described	Breast cancer	Bartoschek et al. (2018)
dCAFs	PDGFRα, SCRG1, SOX9, SOX10	Breast cancer	Bartoschek et al. (2018)
CD10+/GPR77 + CAF	α-SMA, PDGFRβ, FAP, FSP1, Collagen1, CD10, GPR77, IL-6	Breast cancer	Hu et al. (2021)
CAF-A	MMP2, DCN, COL1A2	Colorectal cancer	Li et al. (2017)
CAF-B	ACTA2, TAGLN, PDGFA	Colorectal cancer	Li et al. (2017)
CD105 + CAF	CD105+, FAP, Ly6cl	Pancreatic cancer	Hutton et al. (2021)
CD105-CAF	CD105-, ITG26, CD74, MHC-II	Pancreatic cancer	Hutton et al. (2021)
CD16 + CAF	CD16^+^, αSMA+, FAP, CD10−, GPR77−	Breast cancer	Liu et al. (2022)
SLCWA1 + irCAF	SLC14A1	Bladder cancer	Ma et al. (2022)

### 3.1 CAF heterogeneity and MDSCs

**FIGURE 2:**
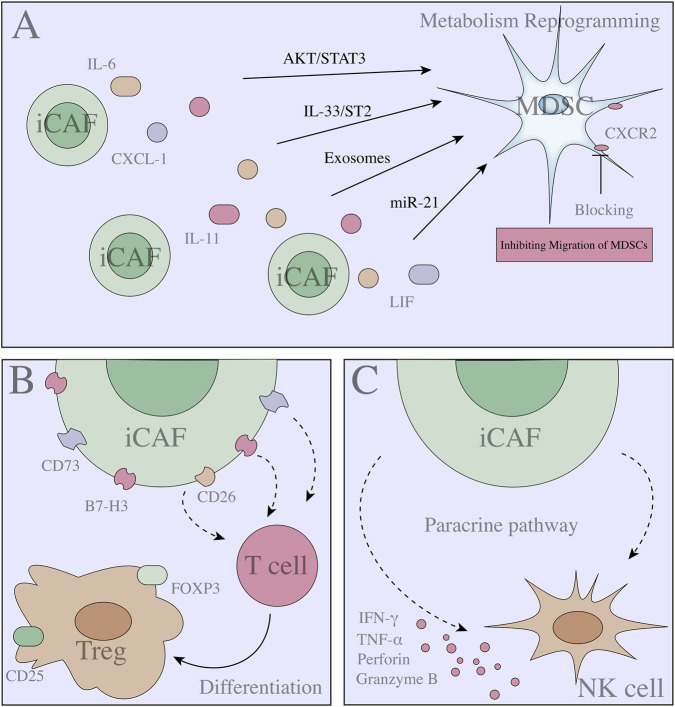
Association between heterogeneous populations of CAFs and immune cells and mechanisms (Part 1). **(A)** iCAFs release IL-6, IL-11, CXCL-1 and LIF into the TME, reshaping MDSC function and inducing metabolic reprogramming. In addition, iCAFs directly induce MDSC AKT/STAT3 and IL33/ST2 signalling to regulate MDSC function. **(B)** iCAFs induce T-cell differentiation. **(C)** iCAFs possess strongly secretion activity, that can release IFN-γ, TNF-α, Perforin and Granzyme B to regulate NK cell activity.

Before single-cell technology became available, investigators gradually identified inflammatory CAF subtypes (iCAFs) and distinguished them from other subtypes on the basis of their αSMA expression (α-SMA^low^, IL-6^high^) (Ohlund et al., 2017) (Figure 2). iCAFs have a unique transcriptomic profile and can secrete inflammatory mediators such as IL-6, IL-11 and LIF and participate in the activation of IL-1 signalling (Biffi and Tuveson, 2021). In addition, iCAFs release large amounts of IL-6, IL-8, CXCL1, CXCL2, CXCL12 and CCL2. Studies have shown that CCL2 is released by a variety of cells in the tumour microenvironment and that CAFs are important for the release of CCL2, which controls the recruitment of monocytes and myeloid suppressor cells (MDSCs) (Chen et al., 2017; Murdoch et al., 2004). CCL2 promotes MDSC recruitment and leads to decreased efficacy of immune checkpoint inhibitors and even drug resistance (Chen et al., 2017; Murdoch et al., 2004). In addition, CAFs release other chemokines, such as CXCL1, which is involved in MDSCs recruitment. Notably, CXCL1 appears to be released exclusively by iCAFs (Ohlund et al., 2017; Kumar et al., 2017). In *in vivo* solid tumour models, CXCL1 produced by CAFs enables a large number of MDSCs to infiltrate the TME, inhibiting immune cell function and promoting tumour progression (Ohlund et al., 2017; Kumar et al., 2017). Interestingly, specifically blocking CXCR2 (a CXCL1 receptor) prevented the migration and infiltration of MDSCs into TME, thereby improving the therapeutic effect (Ohlund et al., 2017; Kumar et al., 2017). In addition, CAFs can also release interleukin 6 (mainly released by iCAFs) to promote MDSC differentiation and thus exert immunosuppressive effects in pancreatic cancer (Deng et al., 2017; Mace et al., 2013). iCAFs seem to be the predominant group of CAFs that release inflammatory mediators (Deng et al., 2017; Mace et al., 2013). Certainly, some functional differences were detected among different heterogeneous CAF groups. In intrahepatic cholangiocarcinoma (ICC), CAFs induce excessive activation of 5-LO metabolism in CD33^+^ MDSCs through IL-33/ST2/AKT/STAT3 and IL-6/IL-6R/AKT/STAT3 signalling. This results in the accumulation of LTB4, which activates BLT2 to increase tumour cell stemness via PI3K/AKT/mTOR signalling activation, leading to tumour progression and drug resistance. Importantly, BLT2 antagonists can effectively improve the efficacy of gemcitabine in mouse PDX models (Lin et al., 2022). In oesophageal squamous cell carcinoma, CAFs release interleukin IL-6 and exosome-packaged microRNA-21 (miR-21) to activate transcriptional signal transduction activator 3 (STAT3) through autocrine and paracrine pathways. M-MDSC production is synergistically promoted (Zhao et al., 2021). These studies suggest that the regulation of immune function by CAFs depends not only on the number of MDSCs but also on their effects on differentiation processes and biological processes (such as metabolism).

Many studies have shown that CAFs are highly heterogeneous, that different groups of CAFs have different functions, and that these groups may participate in different aspects of tumour regulation through synergistic effects (Affo et al., 2021; Bhattacharjee et al., 2021; Miyazaki et al., 2020; Guo et al., 2023). In a preclinical model, blocking TGF-β, a molecule secreted by myCAFs, also led to a significant reduction in the number of MDSCs in TME (Martin et al., 2020). With the development of single-cell sequencing technology and spatial transcriptome sequencing, these studies provide a good theoretical basis for subsequent research as well as promising prospects for personalized medicine. The modes of interaction and correlation between cells are potential directions for further exploration tumour-targeted therapy. Targeting these CAF-related active factors to affect the function of MDSCs and reverse tumour immunosuppression may provide a better method for future immunotherapy.

**FIGURE 3:**
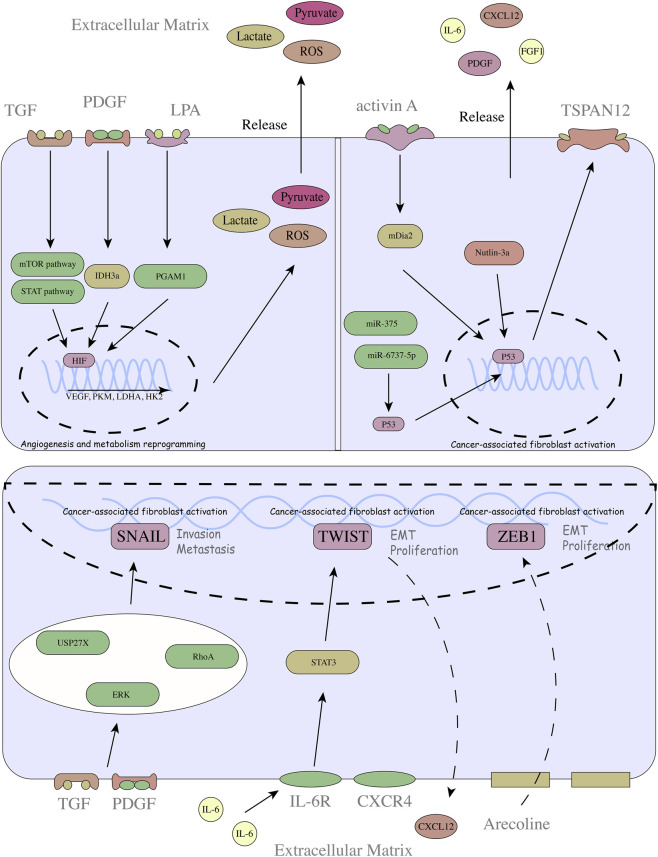
Association between heterogeneous populations of CAFs and immune cells and mechanisms (Part 2).

In a single-cell sequencing study of HCC, CAFs were classified into five types: 1. Vascular CAFs (vCAFs) characterized by the expression of microvasculature genes (MYH11, MUSTN1, and MCAM); 2. Matrix CAFs (mCAFs) with low α-SMA expression and high extracellular matrix (ECM) signatures; 3. Lipid processing (lp)-mCAFs (lpmCAFs) associated with ECM, cholesterol metabolism, fatty acid metabolism and reactive oxygen species (ROS) pathways; 4. Lipid-processing CAFs (lpCAFs) associated with protein-lipid complex remodelling and fatty acid metabolism; and 5. Antigen-presenting CAFs (apCAFs) characterized by chemokine-related genes (Zhu et al., 2023). Among them, the distribution of lpmCAFs (CD36^+^) was highly consistent with that of MDSCs. As a surface membrane protein, CD36 can mediate oxLDL uptake, thereby affecting the secretion of MIF by lpmCAF cells through lipid peroxidation/p38/CEBPs, and MIF promotes an MDSC-related immunosuppressive environment and enhances tumour stemness through IL-6/STAT3 signalling (Zhu et al., 2023). These findings suggest that CD36 expression is population differential and that this unique expression pattern provides an important basis for the secretory function of CAFs (Figure 3). Notably, previous studies have reported the malignant effect of CD36 on pancreatic cancer and hepatocellular carcinoma (Tanase et al., 2020; Yang et al., 2022). In combination with the results of current single-cell sequencing, the mechanism through which high CD36 expression is involved in the regulation of tumour malignancy was initially clarified.

### 3.2 CAF heterogeneity and Tregs

In breast cancer (BC), Costa et al. reported that in the fibroblast subpopulation CAF1-S1, which is characterized by immunosuppression and high expression of αSMA (iCAF), iCAFs secrete high levels of CXCL12, promoting CD4^+^ CD25^+^ T-cell recruitment and infiltration. In addition, B7-H3, CD73 and CD26 overexpression induces T-cell differentiation into CD25^high^ FOXP3^high^ Tregs *in vitro* (Costa et al., 2018). These findings are consistent with previous results showing that targeting CXCL12 combined with PD-L1 immunotherapy has a synergistic effect on preventing pancreatic cancer progression (Feig et al., 2013). Interestingly, CXCL12 is considered a marker of iCAFs in PDAC (Elyada et al., 2019). Similar to the results mentioned earlier, CAFs are highly heterogeneous and functionally different among different populations that are jointly involved in tumour progression through synergistic or antagonistic effects. Surprisingly, Kieffer et al. used single-cell analysis to demonstrate that CAFs consist of eight distinct subpopulations in BC tissues. The authors further show that Treg differentiation is mediated by the myCAF subcluster (ECM-myCAF) rather than iCAFs and that CD4^+^ CD25^+^ T cells, in turn, also influence myCAF phenotype (Kieffer et al., 2020). These findings suggest that identifying the phenotype of CAF subpopulations and evaluating their function are essential for evaluating patient outcomes and antitumour efficacy.

In addition, CAFs are important functional cells that secrete TGF-β. In addition to promoting Treg differentiation, CAF-derived TGF-β can directly inhibit cytotoxic T cells (thus hindering antitumour immunity), and TGF-β has been shown to increase PD-1 expression in tumours and trigger Treg accumulation (Martin et al., 2020; Li et al., 2020; Mariathasan et al., 2018). Targeting TGF-β to improve tumour prognosis is a viable option. It is not only released by one CAF subpopulation but also mediated by other known/unknown subpopulations, and targeted CAF therapy has a largely unknown effect.

### 3.3 CAF heterogeneity and NK cells

In an *in vitro* coculture system, colorectal cancer (CRC)-derived fibroblasts (α-SMA^+^) inhibit the activity of NKs via PGE2 (Li et al., 2013). These cells also inhibit the function of NK cells by reducing the expression of IFN-γ, TNF-α, perforin and granzyme B through the paracrine pathway (Huang Q. et al., 2019). Nociceptor neurons release calcitonin gene-related peptide (CGRP) and nerve growth factor (NGF) to the pancreatic cancer TME and act on CAFs, leading to the inhibition of CAF-related IL-15. Reduced IL-15 expression inhibits NK cell infiltration and cytotoxic function (Wang et al., 2025). Intriguingly, follistatin-like protein 1 (FSTL1)-related CAFs exhibit an immunosuppression phenotype *in vitro*, and CAF-derived FSTL1 upregulates NK cellular NCOA4 expression via the DIP2A-P38 pathway, leading to NK cell ferroptosis (Yao et al., 2023). NK–CAF studies revealed that CAFs with high FSTL1 expression strongly interfere with NK cellular activity. Another study demonstrated a similar mechanism linking CAFs and NK cells: high expression of dickkopf-1 (DKK1) in CAFs *in vitro* inhibits NK cell activity and cytotoxicity by preventing AKT/ERK/S6 phosphorylation (Lee et al., 2025).

In PDAC, NetG1+ CAFs provide nutritional support for malignant cell survival, as shown by *in vitro* assays; in addition, these cells can secrete granulocyte‒macrophage colony-stimulating factor (GM-CSF), IL-1β, IL-6 and IL-8 to directly inhibit (NK) activity (Francescone et al., 2021). Targeting NetG1 or its downstream signals (such as AKT/4E-BP1 and p38/FRA1) can reshape the CAF phenotype to inhibit tumour progression (Francescone et al., 2021). These findings revealed that CAF heterogeneity not only affects the adaptive immune system but also modulates the innate immune system via the paracrine pathway. Senescence-associated secretory-phenotype CAFs (senCAFs) have been identified in human breast cancer, and myofibroblastic p16+ senCAFs produce and reshape the extracellular matrix, decreasing NK cytotoxicity (Ye et al., 2024). Consequently, the heterogeneity of CAFs and NK cells establishes a complex communication mode, representing a potential target for improving clinical treatment.

### 3.4 CAF heterogeneity and T cells

Another CAF subtype, antigen-presenting cancer-associated fibroblasts (apCAFs), has been shown to control Treg differentiation and promote its accumulation in the TME. apCAFs are mesothelial cells that are characterized by the expression of MHC class molecules but lack the expression of conventional costimulatory proteins such as CD80, CD86, and CD40, and apCAFs, which are positively associated with Tregs. However, further experimental evidence is lacking (Huang et al., 2022; Dominguez et al., 2020; Hutton et al., 2021; Kieffer et al., 2020; Mariathasan et al., 2018). Using scRNA-seq, Rolf A et al. reported that apCAFs derived from mesothelial cells from a transcriptional profile perspective can induce naïve CD4^+^ T cells to transform into regulatory T cells; intriguingly, the use of a monoclonal antibody (mAb) against the mesothelial cell marker mesothelin (MSLN Ab) can effectively decrease the transition of mesothelial cells to apCAFs (Huang et al., 2022). In another study, the ability of apCAFs to present model antigens to CD4^+^ T cells *in vitro* was reported, but apCAFs lack the costimulatory molecules required to induce T-cell proliferation. apCAFs may induce CD4^+^ T-cell inactivation or differentiation into Tregs through the expression of MHC class II molecules as decoy receptors, in which case, apCAFs may represent key factors leading to immunosuppression (Elyada et al., 2019).

Fibroblast activation protein (FAP) plays a key role in the immunosuppressive tumour environment. It is currently believed that the high expression of CAFs in FAP can lead to primary drug resistance to immunotherapy (Ohlund et al., 2017; Cremasco et al., 2018; Yang et al., 2016). However, the role of FAP^high^ CAFs in the regulation of immunosuppression in human cancers has not been addressed. In a study on breast cancer, eight CAF subsets were validated using single-cell sequencing, in which the abundance of ECM-myCAFs and TGFβ-myCAFs correlated with the abundance of PD-1 and/or CTLA-4 CD4 T cells. Importantly, ECM-myCAFs could increase PD-1 and CTLA-4 expression on the surface of FOXP3 Tregs, whereas CD4^+^ CD25^+^ T lymphocytes could promote the subsequent transformation of ECM-myCAFs to TGF-myCAFs. These results revealed that numerous regulatory mechanisms exist between CAFs and the immune environment, especially T cells, and that many feedback mechanisms may be critical to tumour immune tolerance (Kieffer et al., 2020). These results suggest that ECM-myCAFs are involved in regulating the subcellular localization of PD-1 and CTLA-4 in FOXP3^+^ T cells, whereas increased CTLA-4 expression on the Treg surface may also serve as a mechanism to bypass PD-1 blockade and promote the persistence of immune exhaustion.

A CAF subtype, defined by FAP expression, has also been shown to promote pancreatic cancer growth through CXCL12 (SDF1) and CCL2 signalling (Feig et al., 2013; Yang et al., 2016). In mouse models, loss of the FAP protein in stromal cells delays the progression of pancreatic cancer. In addition, FAP overexpression is associated with poor outcomes and disease-free survival in PDA patients (Lo et al., 2017). Notably, different populations share similar characteristics; therefore, whether some subsets of CAFs belong to the same population is debated, and model-specific data have been interpreted by different laboratories (Feig et al., 2013; Kieffer et al., 2020; Kraman et al., 2010; Ohlund et al., 2014; Puram et al., 2017).

### 3.5 CAF heterogeneity and macrophages

Although the above studies have demonstrated the functional regulation of CD36 on CAFs, CD36 is highly expressed in metastasis-associated macrophages (MAMs) and is an important regulator of liver metastasis (Yang et al., 2022). Owing to the abnormal metabolic characteristics of tumour cells, the tumour microenvironment is often characterized by hypoxia, decreased pH, increased nitric oxide levels, and increased reactive oxygen species (Leone and Powell, 2020). These abnormal substances provide a rich source of lipid synthesis for tumours (Broadfield et al., 2021). Interestingly, the accumulation of long-chain fatty acids (LCFAs), especially oleic acid, is key to M2 macrophage polarization (Wu et al., 2019). CD36 overexpression may contribute to the abundance of MAMs, which are associated with abnormal metabolites in the TME. These data also provide new insights into targeted therapy. Targeting CD36 may have an effective antitumour effect by inhibiting the function of CAFs and the appearance of M2 macrophages. In addition, CD36 plays an important regulatory role in the function of T cells in tumours and can effectively activate T-cell ferroptosis and impair the antitumour activity of T cells (Ma et al., 2021). Although tumours are highly heterogeneous, the dependence of different cell populations on CD36-associated lipid metabolism in part determines the feasibility of developing therapeutic agents targeting CD36.

Compared with normal tissue, periostin (encoded by POSTN) is an overexpressed stromal cell protein in lung cancer and the production of periostin by CAFs promotes tumour stemness and malignancy (Malanchi et al., 2011; Wei et al., 2021). Recent studies have explored the important role of CAFs and tumour-infiltrating immune cells in immunosuppression (Mao et al., 2021). Secreted phosphorylated protein 1 (SPP1)-related macrophages are a ubiquitous group of immunosuppressive TAMs that act as key mediators during the malignant progression of lung cancer (Cheng et al., 2021; Yang et al., 2021). The abundance of SPP1^+^ TAMs is associated with poor prognosis (Cheng et al., 2021; Yang et al., 2021). Studies have shown that POSTN^+^ CAFs are located mainly around or near tumour nests and in proximity to SPP1^+^ macrophages in lung cancer, where they contribute to ECM remodelling and immunosuppression. POSTN^+^ CAFs had less T-cell infiltration in the tumour area and exhibited a depleted phenotype. This distribution indicates that POSTN^+^ CAFs play important roles in the regulation of macrophage recruitment and T-cell recruitment and function (Chen et al., 2023). These results clarify the potential link between POSTN^+^ CAFs and macrophages and T cells, potentially providing new insights for POSTN-targeting therapy. Similarly, in another gastric cancer study, POSTN^+^ CAFs recruited more CD163^+^ macrophages to the microenvironment through the activation of macrophage AKT signalling, leading to immune checkpoint blockade (ICB) failure (You et al., 2023). It is important to considered macrophage heterogeneity along with CAF heterogeneity, as this recruitment effect may not be specific to all macrophage types.

## 4 CAF heterogeneity and tumour immunotherapy

Because only a small proportion of patients with pancreatic cancer respond to clinical treatment, one study used single-cell transcriptomes to map CAF heterogeneity in an animal model of pancreatic ductal adenocarcinoma (PDAC) and identified a population of CAFs that are regulated by TGFβ and express the leucine-rich repeat 15 (LRRC15) protein. LRRC15^+^ CAFs are distributed mainly around islets but are not detected in normal pancreatic tissue. In addition, clinical trials of immunotherapy in more than 600 patients have shown that an increased proportion of LRRC15^+^ CAFs is associated with poor efficacy of anti-PD-L1 treatment (Dominguez et al., 2020). LRRC15 is a member of the Leu-rich repeat (LRR) superfamily. The protein family is closely related to a variety of biological cellular functions and is involved in many biological processes, such as cell invasion, motility, and autophagy (Bella et al., 2008; Purcell et al., 2018). LRRC15 is specifically distributed in the regulation of cancer because it tends to be highly expressed in cancer-associated stromal fibroblasts but has minimal effect on normal physiological tissues, and its overexpression promotes tumour-related metastasis (Bella et al., 2008; Purcell et al., 2018). On the basis of the current research results, targeting LRRC15 clearly represents an effective therapeutic measure to improve tumour immunotherapy.

SLC14A1 gene encodes urea transporter type b (UT-B), which promotes the passive transport of urea on the cell membrane. Recently, it has been found to be closely related to human malignant tumours (Shayakul and Hediger, 2004). SLC14A1 expression in malignant prostate cancer tissues is significantly lower than that in benign prostate cancer tissues. In contrast, SLC14A1 expression in the castration group is significantly increased, suggesting that SLC14A1 expression is regulated by androgen (Vaarala et al., 2012). This finding may explain, in part, the sex difference in the incidence of some tumours. Although these studies have revealed a regulatory role of SLC14A1 in tumours, it is unclear whether changes in SLC14A1 expression occur globally or in a specific cell population because of the high heterogeneity of tumour tissues.

A recent study attempted to elucidate this mechanism using single-cell sequencing. CAFs in bladder cancer tissues play important roles in tumour treatment response and patient prognosis. CAFs show phenotypic and functional heterogeneity and vary greatly in tumours of different tissue origins. Single-cell RNA sequencing revealed a CAF subset characterized by SLC14A1 overexpression. SLC14A1^+^ CAFs increase the stemness of BC cells through paracrine WNT5A. Inhibition of SLC14A1^+^ CAF formation by targeting STAT1 or STING sensitizes tumour cells to chemotherapy. The high proportion of SLC14A1^+^ CAFs is key to the poor prognosis of patients with breast cancer, and this type of breast cancer has a poor response rate to immunotherapy (Ma et al., 2022). These results confirmed that the changes in SLC14A1 expression were likely caused by changes in a subset of cell populations. In addition, this study elucidated the regulatory mechanism of interferon on the emergence of SLC14A1-high CAF population, which is of interest for subsequent clinical targeted therapy. However, several limitations need to be overcome. Specifically, SLC14A1 expression is universal and is widely present even in normal physiological tissues, and targeted therapy based on this would potentially lead to unexpected toxicity reactions.

High-grade serous ovarian cancer (HGSOC) is among the main types of ovarian cancer; early diagnosis of HGSOC is difficult, and it is often found at an advanced stage. With the development of single-cell sequencing technology, studies have attempted to explore the heterogeneity of CAFs in an effort to elucidate the key driving tumour drug resistance,. With the clinical application of chemotherapy, the proportion of ANTXR1^−^ iCAFs gradually increases, whereas the proportion of ANTXR1^+^ myCAFs gradually decreases (Licaj et al., 2024). These findings indicate that CAFs gradually transition from an inflammatory phenotype to a myofibroblast phenotype in the HGSOC microenvironment after chemotherapy. However, the current understanding of ANTXR1 remains superficial and needs to be further explored. Similarly, iCAF levels are significantly increased after treatment for pancreatic and colorectal cancer (Zhang J. et al., 2022; Nicolas et al., 2022). Another scRNA-seq analysis of ovarian cancer revealed that chemotherapy induced reprogramming of CAFs to an inflammatory state (Zhang K. et al., 2022). These findings suggest that inflammation-associated CAFs play a similar role in multiple tumours after chemotherapy. Interestingly, compared with platinum-resistant HGSOC patients with a low proportion of myCAFs, those with a high proportion of myCAFs after chemotherapy experienced earlier recurrence and shorter survival, suggesting the importance of targeting myCAFs after chemotherapy (Licaj et al., 2024). This study revealed that the reduction in the myCAF content and increase in iCAF proportion after chemotherapy were mainly found in chemotherapy-sensitive patients, not in nonresponsive patients. Combined with the results of the above studies, it is clear that functional subsets still exist in the myCAFs/iCAFs and other cell populations of CAFs, highlighting the importance of cellular function annotation.

## 5 Diagnosis and prognosis according to CAF heterogeneity

According to previous studies, a high matrix ratio or abnormal α-SMA expression is usually associated with poor clinical prognosis for cancer patients (Erkan et al., 2008). However, knocking down α-SMA expression can induce immunosuppression and decrease the survival rate in mice (Ozdemir et al., 2014). Therefore, more accurate diagnoses and therapeutic molecules are crucial for cancer treatment. A recent study revealed that IL-33 and CXCL3 expressions are negatively associated with the survival time of pancreatic cancer patients (Sun et al., 2022). These findings indicate that IL-33 and CXCL3 can serve as diagnostic markers in pancreatic cancer patients. In addition, elevated levels of LRRC15+ CAFs in tumour tissues inhibit immunotherapy efficacy and indicate poor outcomes for tumour patients (Dominguez et al., 2020). Consequently, LRRC15+ CAFs can serve as a prognostic marker to predict immune checkpoint inhibitor efficacy. A recent study using *in situ* hybridization technology revealed that Meflin + CAFs were associated with a favourable prognosis for patients (Mizutani et al., 2019). These findings indicate that evaluating the proportion of Meflin + CAFs can provide a novel method to predict patient prognosis. Similarly, in solid cancer patients, the presence of podoplanin + CAFs is associated with poor outcomes, suggesting that podoplanin is a potentially valuable prognostic marker (Hu et al., 2018). These results revealed diagnostic and prognostic markers that can be used in future clinical evaluation and treatment. In breast cancer, CAF-rich regions are also enriched in a large number of NK cells, and CAFs overexpress NK-binding ligands near these regions (Ben-Shmuel et al., 2025). Consequently, CAFs and their binding ligands can influence outcome and treatment efficiency. DKK1, a highly expression molecule, represents a barrier to antitumour immunity that can serve as an indicator to evaluate immunotherapy efficiency (Lee et al., 2025). Similarly, the release of DKK1 by CAFs can lead to treatment failure and create an immunosuppressive TME (Yin et al., 2025). According to the unique characters of CAFs, developing a novel prognostic marker may provide possible strategy.

## 6 Targeting CAF heterogeneity to improve immunotherapy efficacy

Although gene interference can be achieved *in vitro* using RNA interference and lentivirus technologies, these technologies are difficult to apply in clinical treatment. Therefore, this section introduces new therapies targeting CAF markers, including monoclonal antibodies and small-molecule drugs. With the development of single-cell sequencing technology, although current questions about CAF heterogeneity are being answered, much work is still needed to map the panorama of CAF subsets in different cancers. A positive development is that targeted therapy for molecular markers of cell subsets provides new insights for subsequent clinical work. An intrinsic relationship between TGFβ-associated LRRC15+ CAFs was clearly identified. Notably, subsequent studies have shown that targeting TGFβ combined with PD-1/PD-L1 inhibitors can achieve effective therapeutic effects, potentially caused by inhibition of the effect of the LRRC15 phenotype (Purcell et al., 2018). This treatment was shown to reverse some of the pathological properties of CAFs in breast cancer (Lim et al., 2021). These findings suggest that combined immunotherapy targeting TGF has good therapeutic prospects. However, it is worth considering that this strategy may be appropriate only for solid or highly fibrotic tumours.

In a preclinical study, FAP-2286, a FAP-binding peptide conjugated to a radionuclide chelator, was rapidly and highly taken up by FAP+ tumours but not by normal tissues. In addition, FAP-2286 can effectively prevent malignant tumour progression (Zboralski et al., 2022). However, its effect on immunization has not been evaluated, and further clarification is needed. In another study, a novel antibody–drug conjugate, OMTX705, was synthesized that linked the FAP antibody to a novel cytolysin. OMTX705 alone or in combination with chemotherapy can effectively inhibit tumour progression without the development of tumour resistance and increases CD8^+^ T-cell infiltration in a mouse model. Moreover, OMTX705 can effectively improve the treatment effect of PD-1-resistant solid tumours (Fabre et al., 2020). However, the toxicity of the drugs used for treatment also needs to be considered. Some drugs can cause muscle loss, bone toxicity, cachexia and even death (Tran et al., 2013).

Many biomarkers are considered potential therapeutic targets, and inhibiting CAF function by targeting abnormally expressed molecules is feasible. However, this approach may also trigger uncontrollable systemic reactions. Targeted therapy for the CAF subgroups can lead to an imbalance in the immune system, which may result in unpredictable immune dysfunction. For example, treatment targeting CAFs can lead to an apCAF imbalance, which can interrupt antigen presentation to induce immune escape. Furthermore, in addition to the rash, hypertension, and hypothyroidism reported in the literature, targeted therapy can also cause issues such as lipid metabolism disorders, which in severe cases, may even be life-threatening (Dienstmann et al., 2011). These are all critical points that require further assessment. In the presence of targeted agents, such as EGFR inhibitors, HER-2 inhibitors, and mTOR inhibitors, the main pharmacodynamic toxicities are acneiform rash, hyperglycaemia, and cardiotoxicity (Desage et al., 2024; Gutierrez et al., 2020; Liu and Kurzrock, 2014). Therefore, safety considerations remain important in treatments targeting CAFs. In preclinical studies, targeted treatment strategies based on FAP have been shown to result in manageable adverse events (Prive et al., 2023). However, more evidence needs to be obtained.

## 7 Agents targeting CAFs to improve immunotherapy efficacy

With the in-depth exploration of communication between CAFs and immune cells, strategies for developing targeted drugs for CAFs have been gradually recognized in recent years. As mentioned above, CAFs increase the number of inhibitory immune cells and inhibit the function of effector immune cells, thereby promoting tumour progression in a variety of direct and indirect ways. Therefore, targeting CAF-related immunosuppressive molecules may lead to new prospects for clinical treatment (Table 3: clinical and preclinical studies).

**TABLE 3:** Clinical trials targeting CAF/CAF heterogeneity.

Drugs	Cancer type	Outcome	Clinical trial identifier
Galunisertib + gemcitabine	Pancreatic cancer	Prolonged OS	NCT01373164
SHR‐1701	Cervical cancer	Antitumour activity and controllable safety	NCT03774979
M7824	Solid tumours	Antitumour activity and controllable safety	NCT02517398
IPI‐926 + gemcitabine	Pancreatic cancer	Not well	NCT01130142
Vismodegib + gemcitabine	Pancreatic cancer	Not well	NCT01064622
Defactinib plus pembrolizumab plus gemcitabine	Pancreatic cancer	Tolerable	NCT02546531
Galunisertib + sorafenib	Hepatocellular carcinoma	Showed acceptable safety and prolonged OS	NCT01246986
Sonidegib + docetaxel	Triple‐negative advanced breast cancer	Antitumour activity	NCT02027376
Balixafotide + eribulin	HER2‐negative metastatic breast cancer	Antitumour activity and controllable safety	NCT01837095
Galunisertib + neoadjuvant chemotherapy	Rectal cancer	Improved the complete response rate with acceptable toxicity	NCT02688712
Vismodegib + gemcitabine plus nab‐paclitaxel	Pancreatic cancer	Not well	NCT01088815
BL‐8040 + pembrolizumab and chemotherapy	Pancreatic cancer	Expanded the benefit of chemotherapy	NCT02826486
Plerixafor + bortezomib	Myeloma	Improved the objective response rate with acceptable toxicity	NCT00903968
Galunisertib + durvalumab	Pancreatic cancer	Tolerable	NCT02734160
Cabiralizumab + nivolumab	Pancreatic cancer	Safety and objective response rate	NCT04191421
Tocilizumab	Epithelial ovarian cancer	Antitumour immunity and provides survival benefits	NCT01637532

### 7.1 All-trans retinoic acid (ATRA)

In normal tissues, PSCs store retinoic acid (RA), a metabolite of vitamin A. Once PSCs are activated, the expression of RA is rapidly reduced, resulting in the acquisition of an activated myofibroblast phenotype (Froeling et al., 2011; Huang et al., 2016). In the absence of vitamin A, stellate cell activation persists and accelerates tumour progression in several solid tumours (such as hepatocellular carcinoma (Li X. et al., 2023; Lu et al., 2023) and pancreatic cancer (Froeling et al., 2011; Guan et al., 2014)). Retinoic acid inhibits Wnt signalling and forces pancreatic stellate cells (PSCs) to maintain a quiescent state to slow tumour progression (Froeling et al., 2011). In addition to regulating immune responses through CAFs, RA signalling can directly affect Treg function (Thangavelu et al., 2022) and enhance anti-PD-1 immunotherapy efficacy by regulating OTUD6B deubiquitination (Li L. et al., 2023). RA significantly improved the efficacy of anti-CTLA4/anti-PD-L1 combination therapy in a dose-dependent manner (Tilsed et al., 2022). Mechanistically, RA promotes the formation of a tumour inflammatory environment, including the infiltration of inflammatory macrophages. However, notably, in haematological malignancies, all-trans retinoic acid plays the opposite role, reducing B7-H6 expression through the c-Myc signalling pathway to promote leukaemia resistance to NK cytotoxicity (Cao et al., 2021). Although it may play opposite roles in different tumours, it is worth recognizing that RA-related signalling plays important roles in CAFs and immune cells. Improved utilization of these features may provide promising methods for subsequent immunotherapy. At present, phase I clinical trials are being carried out (ATRA combined with conventional chemotherapy drugs) (Kocher et al., 2020).

### 7.2 Vitamin D receptor (VDR)

The poor prognosis and drug resistance of pancreatic ductal adenocarcinoma (PDA) are attributed to the tumour microenvironment (Mahadevan and Von Hoff, 2007; Rasanen and Vaheri, 2010; Shimoda et al., 2010). The transformation of pancreatic stellate cells (PSCs) from a quiescent state to an activated state drives the severe stromal response that characterizes PDA (Mahadevan and Von Hoff, 2007). Studies have shown that the vitamin D receptor (VDR) is abnormally expressed in the stroma of human pancreatic tumours. Calcipotriol acts as a ligand of VDR and inhibits inflammation and fibrosis in pancreatitis and in the tumour stroma (Sherman et al., 2014). In pancreatic cancer cells, calcipotriol inhibits the ability of CAFs to promote tumour progression but can also reduce the killing function of T cells (Gorchs et al., 2020). Therefore, the activation of VDR signalling appears to have a dual function in pancreatic cancer. This largely explains the differences in the prognosis of individuals with different tumours after they were after taking vitamin D. Vitamin D and calcium intake for breast cancer were inversely associated with breast cancer risk, whereas some studies reported no association (Chlebowski et al., 2008; Garland et al., 2007; John et al., 1999; Shin et al., 2002). This difference may be related to the physiological state of the patients (premenopausal/postmenopausal) (Ahn et al., 2008; Anderson et al., 2010; Lin et al., 2007). In a cross-sectional study of colorectal cancer, serum 25(OH)D concentrations were inversely associated with adenomas and colonic polyps (Hightower et al., 2017). A meta-analysis revealed that higher circulating 25(OH)D levels reduce mortality in patients with colorectal cancer (Mohr et al., 2015). However, these studies are based on epidemiological surveys, and the specific mechanisms still need to be further explored. Several studies have shown that the cancer-promoting effect of CAFs depends on their strong secretory properties and that vitamin D inhibits the secretion of exosomes containing miR-10a-5p from CAFs, thereby inhibiting tumour progression (Ferrer-Mayorga et al., 2017; Kong et al., 2019). miR-10a-5p is an important regulator of immune cells (Zhang et al., 2019; Madrid-Elena et al., 2022; Xie et al., 2023). These results suggest the important role of vitamin D and VDR signalling in CAFs and immune regulation, which occurs both directly and indirectly and is related to the strong secretory characteristics of CAFs.

### 7.3 Setanaxib

The ROS-producing enzyme NADPH oxidase-4 (NOX4) regulates the differentiation of myofibroblast CAFs in a variety of cancers, and NOX4 inhibition can restore activated CAFs to a quiescent state (Hanley et al., 2018). Studies have shown that inhibition of NOX4 (setanaxib) can precisely reverse CAF differentiation and promote CD8^+^ T-cell infiltration in a CAF-dependent manner, thus enhancing immunotherapy sensitivity (Ford et al., 2020). As a downstream target of TGF-β1, NOX4 inhibition can effectively reverse the activation of CAFs induced by TGF-β1 (Sampson et al., 2018). These results suggest that inhibition of downstream TGF signalling can play the same role, but the effects of NOX4 inhibition on other cell types should be considered.

### 7.4 TGF signalling inhibitors

High TGF-β1 levels are observed in bladder cancer tissues and fibroblast culture media. TGF-β1 is secreted mainly by fibroblasts in tumour tissue. TGF-β1 secreted by CAFs promotes the migration, invasion and EMT of bladder cancer cells through FAP/Versican (Ping et al., 2023). The combination of the TGF-β inhibitor LY2157299 with photothermal therapy can significantly improve the efficacy of CAR-T-cell therapy (Tang et al., 2023). TGF-β1 neutralization enhanced the therapeutic effect of the combination of FOLFOX and anti-PD-1 therapy and induced antigen-specific CD8^+^ T-cell recruitment to tumours. Mechanistically, TGF-β1 regulates CXCL9 and CXCL10 production in a G9a- and EZH2-dependent manner (Vienot et al., 2022). In breast cancer, TGF-beta-activated CAFs were found to be specifically derived from resistant tissues (Rivas et al., 2022). These findings also indicate that the use of TGF inhibitors can effectively block the drug resistance process, thereby enhancing the therapeutic effect. Other studies have shown that ECM remodelling is associated with the aberrant activation of TGF-β signalling in cancer-associated fibroblasts, leading to CAF-related oncogene abnormalities, such as BRAF, SMAD4 and TP53 mutations and MYC amplification, and that a TGF-β inhibitor can effectively increase the efficacy of immune checkpoint inhibitors (ICBs) (Chakravarthy et al., 2018). Tauriello et al. reported that the combination of TGF-β inhibitors with ICBs effectively prevented the emergence of metastases in some ICB-unresponsive colorectal cancer tumours. Combined treatment with a TGF-β inhibitor and an ICB was highly effective at reducing metastatic spread and the number of metastases (Tauriello et al., 2018). On the basis of TCGA RNA-seq data, the adaptive mechanism through which ECM-related genes promote immune evasion and immunotherapy resistance in tumours has been preliminarily identified, and these genes are associated with multiple types of cancer characteristics. Moreover, the differences in the expression of these ECM-related genes are caused mainly by CAFs (Chakravarthy et al., 2018; Yuzhalin et al., 2018).

### 7.5 CXCR4 inhibition

CAFs derived from triple-negative breast cancer (TNBC) promote monocyte recruitment through CXCL12–CXCR4, and inhibition of CXCR4 effectively inhibits the immunosuppressive effect caused by CAF activation (Timperi et al., 2022). At present, the mechanism of CXCR4 and immune regulation by CAFs has not been fully explored, but it is important to recognize that the ability of CAFs to trigger CXCR4 signalling activation is critical for the regulation of immune cell recruitment. In addition, CAF-related CXCR4 activation may also be involved in the regulation of immune cell function. In metastatic biopsies from patients with microsatellite-stable colorectal cancer and pancreatic cancer, continuous infusion of the small-molecule inhibitor AMD3100 (plerixafor) for 1 week significantly inhibited CXCR4 expression and induced a significant immune response (Biasci et al., 2020).

### 7.6 Tocilizumab

iCAFs exhibit inflammatory features and secrete IL-6, which inhibits NK cell activity and promote tumour progression (Carr and Fernandez-Zapico, 2019). IL-6 and TGF-β co-stimulation promotes the differentiation of Th17 cells and the secretion of IL-17A, thereby providing continuous stimulation for CAF activation. Tocilizumab (TOC), a monoclonal antibody against IL-6R, significantly inhibits IL-6 and TGF-β overexpression in fibroblasts, thereby blocking the positive feedback signal of CAF activation and improving the function of CD8^+^ T cells (Fang et al., 2022). In a mouse model, the combination of an IL-6 inhibitor and an anti-PD-L1 antibody effectively increased treatment efficacy and improved overall survival (Long et al., 2017).

### 7.7 Galunisertib

TGF-β shapes CAFs in the TME, promoting matrix stiffness, lamellipodia formation and cell invasion (Stylianou et al., 2018). TGF-β is responsible for the transformation of CAFs to myCAFs; thus, TGF-β can be targeted to prevent tumour progression (Hezel et al., 2012). Compared with gemcitabine, galunisertib is the first oral drug to inhibit TGF-β, and it significantly improves the outcome of patients (Huang H. et al., 2019; Melisi et al., 2018). In addition, the combination of galunisertib and an anti-PD-L1 antibody (durvalumab) is safe and effective for the treatment of metastatic pancreatic cancer (Melisi et al., 2021).

### 7.8 Nintedanib

To overcome the link between NK-CAFs, antifibrotic drugs can be used to prevent tumour progression and immunotherapy. The use of nintedanib as an antifibrotic drug (targeting platelet-derived growth factor receptor β (PDGFRβ)) alone cannot achieve ideal efficiency, but the combination of nintedanib with mesothelin (MSLN)-related chimeric antigen receptor-NKs can achieve excellent efficacy in tumour spheroid and xenograft models (Lee et al., 2023). The combined expression of MSLN and PDGFRβ in the local tumour area is associated with poor clinical outcomes (Lee et al., 2023). This provides a potential marker for evaluating prognosis and therapeutic outcomes.

## 8 Future perspectives

The impact of CAF heterogeneity on tumour progression is profound, and current studies clearly demonstrate that different groups of heterogeneous CAFs can participate in tumour occurrence, development, immune cell infiltration and even drug resistance. Bidirectional communication between CAFs and other components of the TME further promotes tumour functional heterogeneity, reshaping disease progression and treatment response. Although research linking CAF subgroups to the TME is limited, increasing evidence has clarified that the CAF population can shape and conquer the TME, inducing multicompartment metaprogram, tumour progression, and acquired drug resistance in tumours. In addition, with an in-depth understanding of the cross-linking between CAFs and the immune system, emerging evidence has revealed the potential mechanisms involved in tumour immune inhibition, and this approach has gradually become a promising strategy for tumour intervention. Different CAF groups may have similar characteristics and functions. Therefore, it is particularly important to elucidate the role of CAFs in different tumours. In addition, it should also be considered that different CAF subsets can interconvert, which is crucial for changes in the proportion of CAF subsets. Therefore, the origin of CAF subsets should be clarified, as this information is crucial for subsequent targeted therapy. In this situation, it is of utmost importance to gain a thorough understanding of the heterogeneity of CAFs, clarify the functional diversity of heterogeneous groups, and thereby propose more diverse and effective targets. Single-cell omics and spatial transcriptomics analyses have provided valuable approaches for clarifying the heterogeneity of CAFs, and these technologies have profoundly affected subsequent basic-to-clinical translation (Su et al., 2024; Van de Sande et al., 2023; Zhang et al., 2024). In the future, applying technological methods to CAF research could provide unprecedented insights. Although challenges still exist, continuing to clarify the molecular characteristics and functions of CAF subtypes is crucial for the development of new cancer treatment methods.
